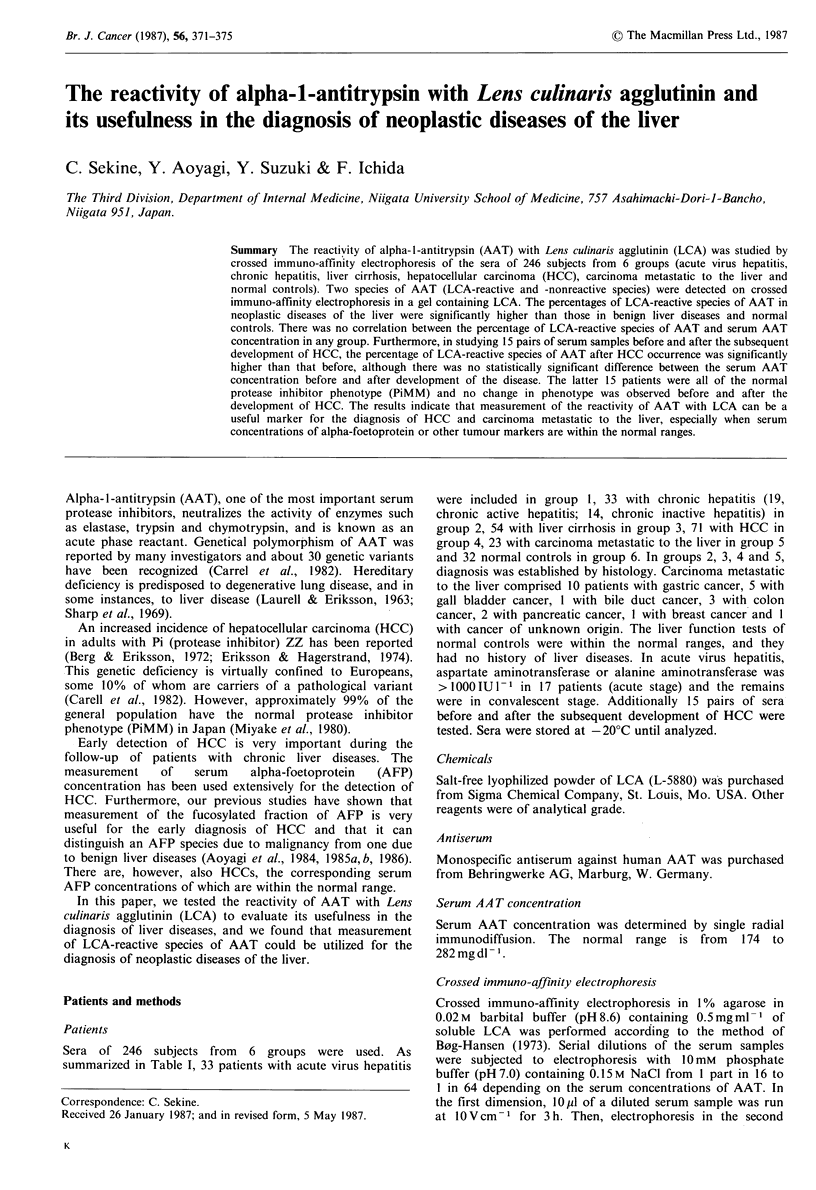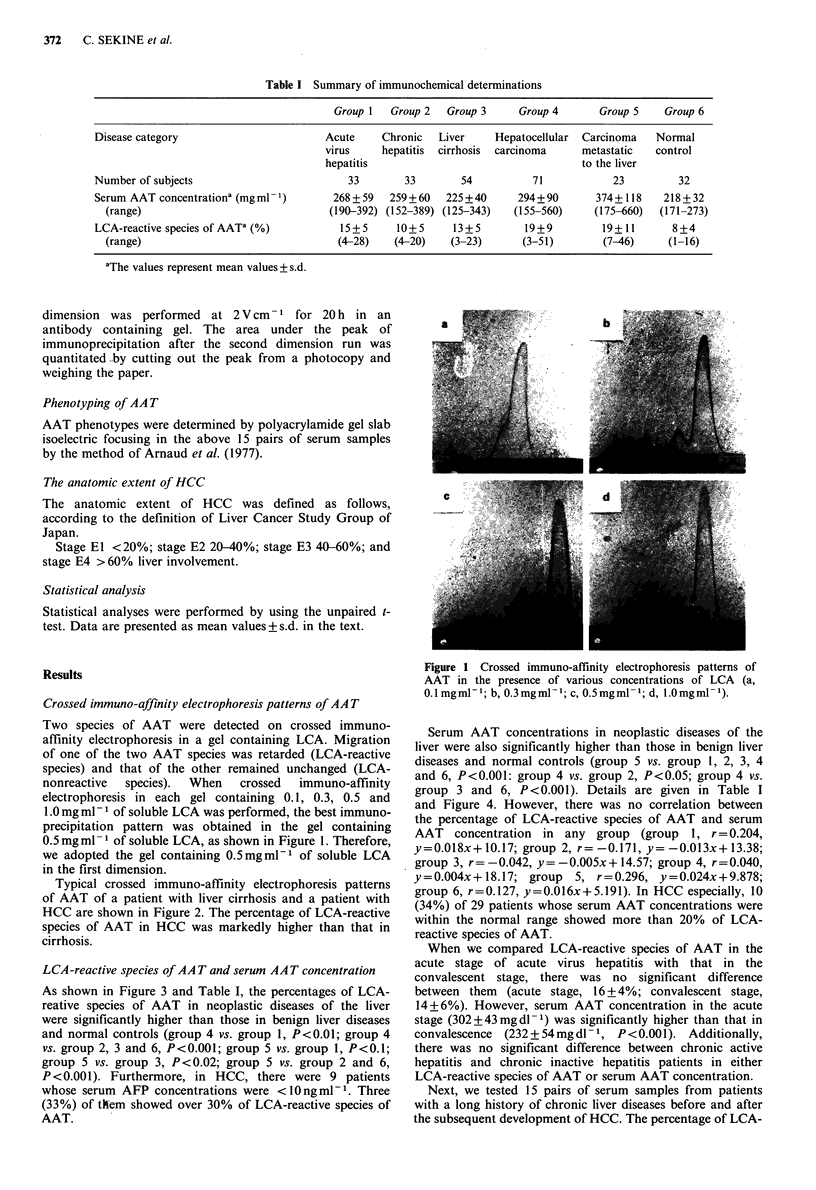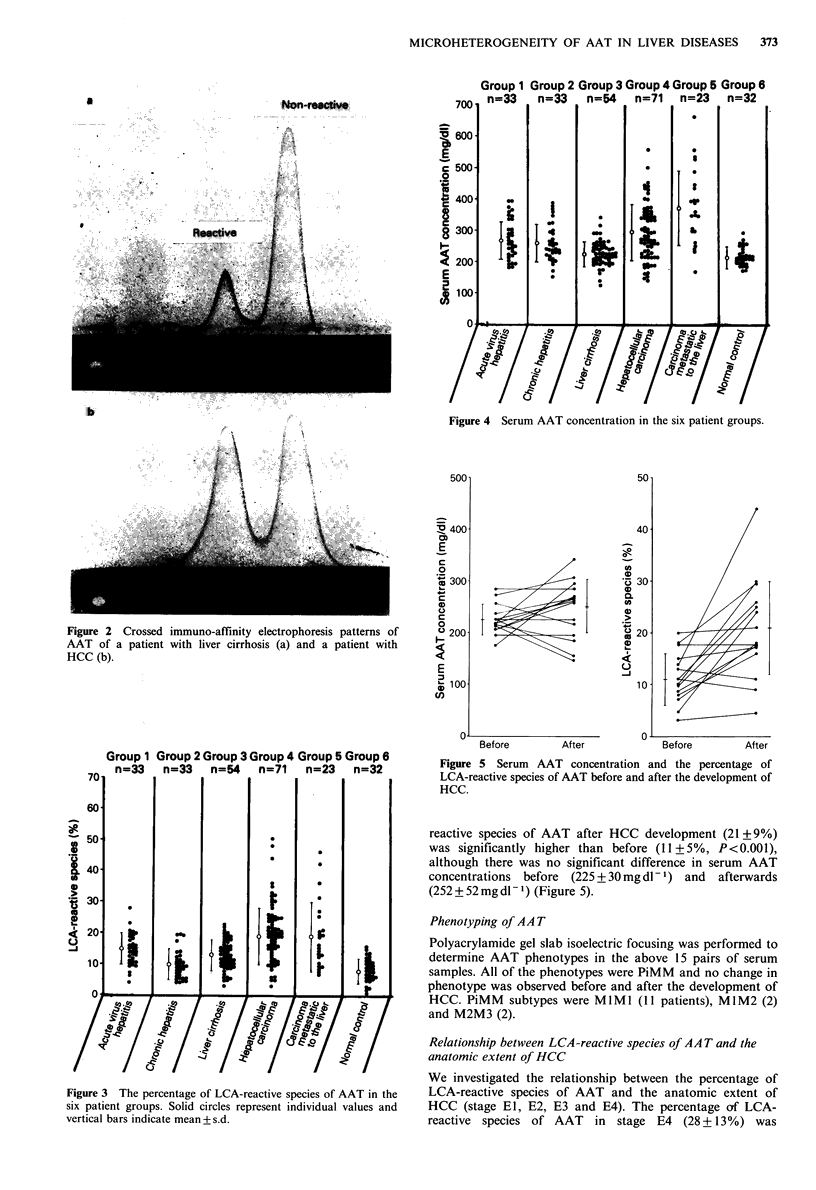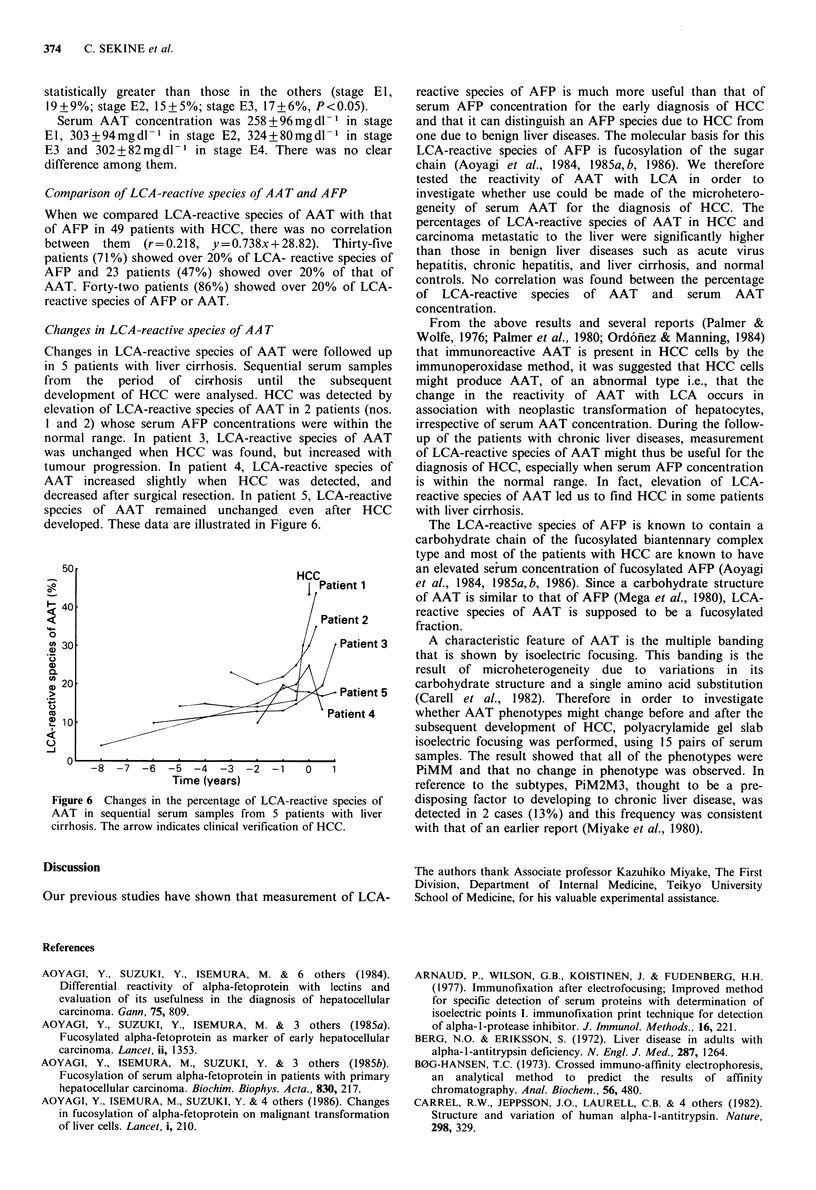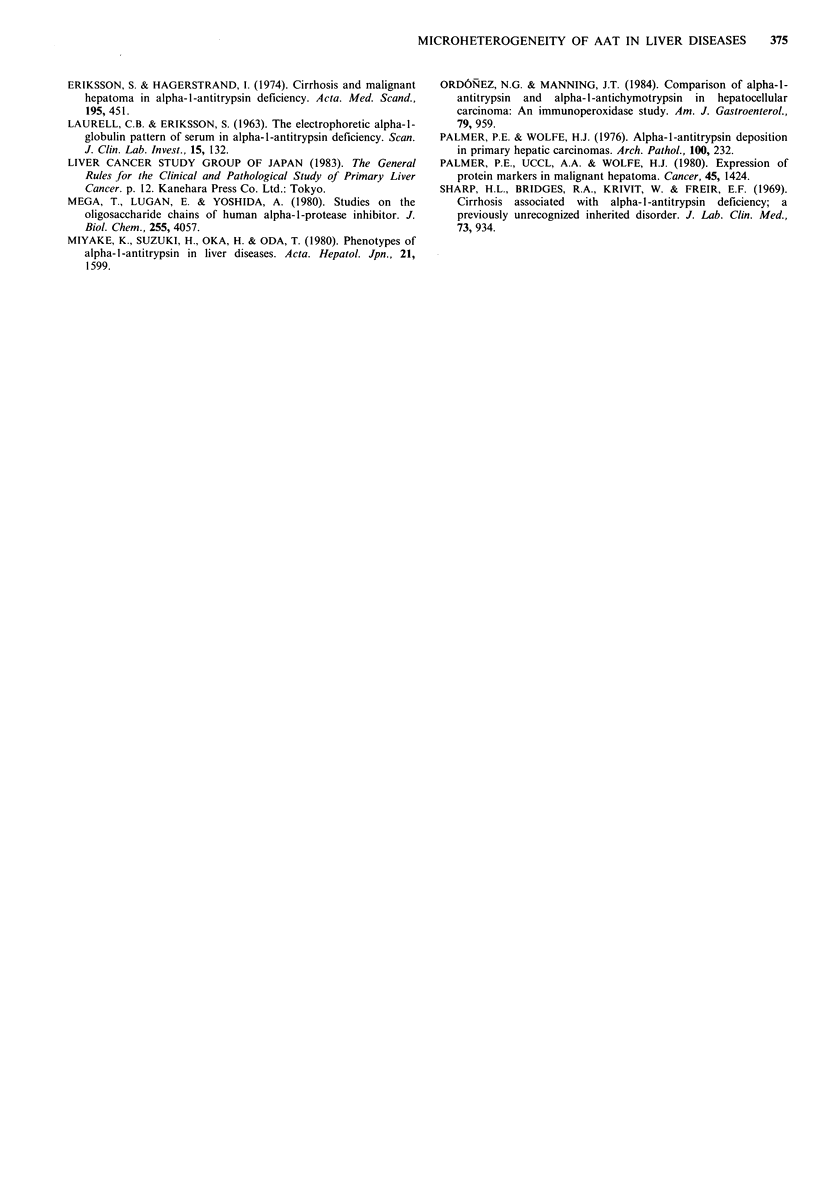# The reactivity of alpha-1-antitrypsin with Lens culinaris agglutinin and its usefulness in the diagnosis of neoplastic diseases of the liver.

**DOI:** 10.1038/bjc.1987.205

**Published:** 1987-09

**Authors:** C. Sekine, Y. Aoyagi, Y. Suzuki, F. Ichida

**Affiliations:** Department of Internal Medicine, Niigata University School of Medicine, Japan.

## Abstract

**Images:**


*Br. J. Cancer* (1987), **56**, 371–375 © The Macmillan Press Ltd., 1987

### The reactivity of alpha-1-antitrypsin with *Lens culinaris* agglutinin and its usefulness in the diagnosis of neoplastic diseases of the liver

C. Sekine, Y. Aoyagi, Y. Suzuki & F. Ichida

*The Third Division, Department of Internal Medicine, Niigata University School of Medicine, 757 Asahimachi-Dori-1-Bancho, Niigata 951, Japan.*

**Summary** The reactivity of alpha-1-antitrypsin (AAT) with *Lens culinaris* agglutinin (LCA) was studied by crossed immuno-affinity electrophoresis of the sera of 246 subjects from 6 groups (acute virus hepatitis, chronic hepatitis, liver cirrhosis, hepatocellular carcinoma (HCC), carcinoma metastatic to the liver and normal controls). Two species of AAT (LCA-reactive and -nonreactive species) were detected on crossed immuno-affinity electrophoresis in a gel containing LCA. The percentages of LCA-reactive species of AAT in neoplastic diseases of the liver were significantly higher than those in benign liver diseases and normal controls. There was no correlation between the percentage of LCA-reactive species of AAT and serum AAT concentration in any group. Furthermore, in studying 15 pairs of serum samples before and after the subsequent development of HCC, the percentage of LCA-reactive species of AAT after HCC occurrence was significantly higher than that before, although there was no statistically significant difference between the serum AAT concentration before and after development of the disease. The latter 15 patients were all of the normal protease inhibitor phenotype (PiMM) and no change in phenotype was observed before and after the development of HCC. The results indicate that measurement of the reactivity of AAT with LCA can be a useful marker for the diagnosis of HCC and carcinoma metastatic to the liver, especially when serum concentrations of alpha-foetoprotein or other tumour markers are within the normal ranges.

Alpha-1-antitrypsin (AAT), one of the most important serum protease inhibitors, neutralizes the activity of enzymes such as elastase, trypsin and chymotrypsin, and is known as an acute phase reactant. Genetical polymorphism of AAT was reported by many investigators and about 30 genetic variants have been recognized (Carrel *et al.*, 1982). Hereditary deficiency is predisposed to degenerative lung disease, and in some instances, to liver disease (Laurell & Eriksson, 1963; Sharp *et al.*, 1969).

An increased incidence of hepatocellular carcinoma (HCC) in adults with Pi (protease inhibitor) ZZ has been reported (Berg & Eriksson, 1972; Eriksson & Hagerstrand, 1974). This genetic deficiency is virtually confined to Europeans, some 10% of whom are carriers of a pathological variant (Carell *et al.*, 1982). However, approximately 99% of the general population have the normal protease inhibitor phenotype (PiMM) in Japan (Miyake *et al.*, 1980).

Early detection of HCC is very important during the follow-up of patients with chronic liver diseases. The measurement of serum alpha-foetoprotein (AFP) concentration has been used extensively for the detection of HCC. Furthermore, our previous studies have shown that measurement of the fucosylated fraction of AFP is very useful for the early diagnosis of HCC and that it can distinguish an AFP species due to malignancy from one due to benign liver diseases (Aoyagi *et al.*, 1984, 1985*a*,*b*, 1986). There are, however, also HCCs, the corresponding serum AFP concentrations of which are within the normal range.

In this paper, we tested the reactivity of AAT with *Lens culinaris* agglutinin (LCA) to evaluate its usefulness in the diagnosis of liver diseases, and we found that measurement of LCA-reactive species of AAT could be utilized for the diagnosis of neoplastic diseases of the liver.

### Patients and methods

### *Patients*

Sera of 246 subjects from 6 groups were used. As summarized in Table I, 33 patients with acute virus hepatitis were included in group 1, 33 with chronic hepatitis (19, chronic active hepatitis; 14, chronic inactive hepatitis) in group 2, 54 with liver cirrhosis in group 3, 71 with HCC in group 4, 23 with carcinoma metastatic to the liver in group 5 and 32 normal controls in group 6. In groups 2, 3, 4 and 5, diagnosis was established by histology. Carcinoma metastatic to the liver comprised 10 patients with gastric cancer, 5 with gall bladder cancer, 1 with bile duct cancer, 3 with colon cancer, 2 with pancreatic cancer, 1 with breast cancer and 1 with cancer of unknown origin. The liver function tests of normal controls were within the normal ranges, and they had no history of liver diseases. In acute virus hepatitis, aspartate aminotransferase or alanine aminotransferase was $>1000\,IU\,l^{-1}$ in 17 patients (acute stage) and the remains were in convalescent stage. Additionally 15 pairs of sera before and after the subsequent development of HCC were tested. Sera were stored at $-20°C$ until analyzed.

### *Chemicals*

Salt-free lyophilized powder of LCA (L-5880) was purchased from Sigma Chemical Company, St. Louis, Mo. USA. Other reagents were of analytical grade.

### *Antiserum*

Monospecific antiserum against human AAT was purchased from Behringwerke AG, Marburg, W. Germany.

### *Serum AAT concentration*

Serum AAT concentration was determined by single radial immunodiffusion. The normal range is from 174 to $282\,mg\,dl^{-1}$.

### *Crossed immuno-affinity electrophoresis*

Crossed immuno-affinity electrophoresis in 1% agarose in 0.02 M barbital buffer (pH 8.6) containing $0.5\,mg\,ml^{-1}$ of soluble LCA was performed according to the method of Bøg-Hansen (1973). Serial dilutions of the serum samples were subjected to electrophoresis with 10 mM phosphate buffer (pH 7.0) containing 0.15 M NaCl from 1 part in 16 to 1 in 64 depending on the serum concentrations of AAT. In the first dimension, 10 $\mu l$ of a diluted serum sample was run at $10\,V\,cm^{-1}$ for 3 h. Then, electrophoresis in the second

Correspondence: C. Sekine.
Received 26 January 1987; and in revised form, 5 May 1987.

**Table I** Summary of immunochemical determinations

| | *Group* 1 | *Group* 2 | *Group* 3 | *Group* 4 | *Group* 5 | *Group* 6 |
|---|---|---|---|---|---|---|
| Disease category | Acute virus hepatitis | Chronic hepatitis | Liver cirrhosis | Hepatocellular carcinoma | Carcinoma metastatic to the liver | Normal control |
| Number of subjects | 33 | 33 | 54 | 71 | 23 | 32 |
| Serum AAT concentration[a] ($mg\,ml^{-1}$) | 268±59 | 259±60 | 225±40 | 294±90 | 374±118 | 218±32 |
| (range) | (190–392) | (152–389) | (125–343) | (155–560) | (175–660) | (171–273) |
| LCA-reactive species of AAT[a] (%) | 15±5 | 10±5 | 13±5 | 19±9 | 19±11 | 8±4 |
| (range) | (4–28) | (4–20) | (3–23) | (3–51) | (7–46) | (1–16) |

[a]The values represent mean values±s.d.

dimension was performed at $2\,V\,cm^{-1}$ for 20h in an antibody containing gel. The area under the peak of immunoprecipitation after the second dimension run was quantitated by cutting out the peak from a photocopy and weighing the paper.

*Phenotyping of AAT*

AAT phenotypes were determined by polyacrylamide gel slab isoelectric focusing in the above 15 pairs of serum samples by the method of Arnaud *et al.* (1977).

*The anatomic extent of HCC*

The anatomic extent of HCC was defined as follows, according to the definition of Liver Cancer Study Group of Japan.

Stage E1 <20%; stage E2 20–40%; stage E3 40–60%; and stage E4 >60% liver involvement.

*Statistical analysis*

Statistical analyses were performed by using the unpaired *t*-test. Data are presented as mean values±s.d. in the text.

### Results

*Crossed immuno-affinity electrophoresis patterns of AAT*

Two species of AAT were detected on crossed immuno-affinity electrophoresis in a gel containing LCA. Migration of one of the two AAT species was retarded (LCA-reactive species) and that of the other remained unchanged (LCA-nonreactive species). When crossed immuno-affinity electrophoresis in each gel containing 0.1, 0.3, 0.5 and $1.0\,mg\,ml^{-1}$ of soluble LCA was performed, the best immunoprecipitation pattern was obtained in the gel containing $0.5\,mg\,ml^{-1}$ of soluble LCA, as shown in Figure 1. Therefore, we adopted the gel containing $0.5\,mg\,ml^{-1}$ of soluble LCA in the first dimension.

Typical crossed immuno-affinity electrophoresis patterns of AAT of a patient with liver cirrhosis and a patient with HCC are shown in Figure 2. The percentage of LCA-reactive species of AAT in HCC was markedly higher than that in cirrhosis.

*LCA-reactive species of AAT and serum AAT concentration*

As shown in Figure 3 and Table I, the percentages of LCA-reative species of AAT in neoplastic diseases of the liver were significantly higher than those in benign liver diseases and normal controls (group 4 *vs*. group 1, $P<0.01$; group 4 *vs*. group 2, 3 and 6, $P<0.001$; group 5 *vs*. group 1, $P<0.1$; group 5 *vs*. group 3, $P<0.02$; group 5 *vs*. group 2 and 6, $P<0.001$). Furthermore, in HCC, there were 9 patients whose serum AFP concentrations were $<10\,ng\,ml^{-1}$. Three (33%) of them showed over 30% of LCA-reactive species of AAT.

**Figure 1** Crossed immuno-affinity electrophoresis patterns of AAT in the presence of various concentrations of LCA (a, $0.1\,mg\,ml^{-1}$; b, $0.3\,mg\,ml^{-1}$; c, $0.5\,mg\,ml^{-1}$; d, $1.0\,mg\,ml^{-1}$).

Serum AAT concentrations in neoplastic diseases of the liver were also significantly higher than those in benign liver diseases and normal controls (group 5 *vs*. group 1, 2, 3, 4 and 6, $P<0.001$: group 4 *vs*. group 2, $P<0.05$; group 4 *vs*. group 3 and 6, $P<0.001$). Details are given in Table I and Figure 4. However, there was no correlation between the percentage of LCA-reactive species of AAT and serum AAT concentration in any group (group 1, $r=0.204$, $y=0.018x+10.17$; group 2, $r=-0.171$, $y=-0.013x+13.38$; group 3, $r=-0.042$, $y=-0.005x+14.57$; group 4, $r=0.040$, $y=0.004x+18.17$; group 5, $r=0.296$, $y=0.024x+9.878$; group 6, $r=0.127$, $y=0.016x+5.191$). In HCC especially, 10 (34%) of 29 patients whose serum AAT concentrations were within the normal range showed more than 20% of LCA-reactive species of AAT.

When we compared LCA-reactive species of AAT in the acute stage of acute virus hepatitis with that in the convalescent stage, there was no significant difference between them (acute stage, 16±4%; convalescent stage, 14±6%). However, serum AAT concentration in the acute stage ($302\pm43\,mg\,dl^{-1}$) was significantly higher than that in convalescence ($232\pm54\,mg\,dl^{-1}$, $P<0.001$). Additionally, there was no significant difference between chronic active hepatitis and chronic inactive hepatitis patients in either LCA-reactive species of AAT or serum AAT concentration.

Next, we tested 15 pairs of serum samples from patients with a long history of chronic liver diseases before and after the subsequent development of HCC. The percentage of LCA-

**Figure 2** Crossed immuno-affinity electrophoresis patterns of AAT of a patient with liver cirrhosis (a) and a patient with HCC (b).

**Figure 3** The percentage of LCA-reactive species of AAT in the six patient groups. Solid circles represent individual values and vertical bars indicate mean ± s.d.

**Figure 4** Serum AAT concentration in the six patient groups.

**Figure 5** Serum AAT concentration and the percentage of LCA-reactive species of AAT before and after the development of HCC.

reactive species of AAT after HCC development (21 ± 9%) was significantly higher than before (11 ± 5%, $P < 0.001$), although there was no significant difference in serum AAT concentrations before ($225 \pm 30\,mg\,dl^{-1}$) and afterwards ($252 \pm 52\,mg\,dl^{-1}$) (Figure 5).

### *Phenotyping of AAT*

Polyacrylamide gel slab isoelectric focusing was performed to determine AAT phenotypes in the above 15 pairs of serum samples. All of the phenotypes were PiMM and no change in phenotype was observed before and after the development of HCC. PiMM subtypes were M1M1 (11 patients), M1M2 (2) and M2M3 (2).

### *Relationship between LCA-reactive species of AAT and the anatomic extent of HCC*

We investigated the relationship between the percentage of LCA-reactive species of AAT and the anatomic extent of HCC (stage E1, E2, E3 and E4). The percentage of LCA-reactive species of AAT in stage E4 (28 ± 13%) was statistically greater than those in the others (stage E1, 19±9%; stage E2, 15±5%; stage E3, 17±6%, $P<0.05$).

Serum AAT concentration was $258\pm96\,\mathrm{mg\,dl^{-1}}$ in stage E1, $303\pm94\,\mathrm{mg\,dl^{-1}}$ in stage E2, $324\pm80\,\mathrm{mg\,dl^{-1}}$ in stage E3 and $302\pm82\,\mathrm{mg\,dl^{-1}}$ in stage E4. There was no clear difference among them.

*Comparison of LCA-reactive species of AAT and AFP*

When we compared LCA-reactive species of AAT with that of AFP in 49 patients with HCC, there was no correlation between them ($r=0.218$, $y=0.738x+28.82$). Thirty-five patients (71%) showed over 20% of LCA- reactive species of AFP and 23 patients (47%) showed over 20% of that of AAT. Forty-two patients (86%) showed over 20% of LCA-reactive species of AFP or AAT.

*Changes in LCA-reactive species of AAT*

Changes in LCA-reactive species of AAT were followed up in 5 patients with liver cirrhosis. Sequential serum samples from the period of cirrhosis until the subsequent development of HCC were analysed. HCC was detected by elevation of LCA-reactive species of AAT in 2 patients (nos. 1 and 2) whose serum AFP concentrations were within the normal range. In patient 3, LCA-reactive species of AAT was unchanged when HCC was found, but increased with tumour progression. In patient 4, LCA-reactive species of AAT increased slightly when HCC was detected, and decreased after surgical resection. In patient 5, LCA-reactive species of AAT remained unchanged even after HCC developed. These data are illustrated in Figure 6.

**Figure 6** Changes in the percentage of LCA-reactive species of AAT in sequential serum samples from 5 patients with liver cirrhosis. The arrow indicates clinical verification of HCC.

### Discussion

Our previous studies have shown that measurement of LCA-reactive species of AFP is much more useful than that of serum AFP concentration for the early diagnosis of HCC and that it can distinguish an AFP species due to HCC from one due to benign liver diseases. The molecular basis for this LCA-reactive species of AFP is fucosylation of the sugar chain (Aoyagi *et al.*, 1984, 1985*a*,*b*, 1986). We therefore tested the reactivity of AAT with LCA in order to investigate whether use could be made of the microheterogeneity of serum AAT for the diagnosis of HCC. The percentages of LCA-reactive species of AAT in HCC and carcinoma metastatic to the liver were significantly higher than those in benign liver diseases such as acute virus hepatitis, chronic hepatitis, and liver cirrhosis, and normal controls. No correlation was found between the percentage of LCA-reactive species of AAT and serum AAT concentration.

From the above results and several reports (Palmer & Wolfe, 1976; Palmer *et al.*, 1980; Ordóñez & Manning, 1984) that immunoreactive AAT is present in HCC cells by the immunoperoxidase method, it was suggested that HCC cells might produce AAT, of an abnormal type i.e., that the change in the reactivity of AAT with LCA occurs in association with neoplastic transformation of hepatocytes, irrespective of serum AAT concentration. During the follow-up of the patients with chronic liver diseases, measurement of LCA-reactive species of AAT might thus be useful for the diagnosis of HCC, especially when serum AFP concentration is within the normal range. In fact, elevation of LCA-reactive species of AAT led us to find HCC in some patients with liver cirrhosis.

The LCA-reactive species of AFP is known to contain a carbohydrate chain of the fucosylated biantennary complex type and most of the patients with HCC are known to have an elevated serum concentration of fucosylated AFP (Aoyagi *et al.*, 1984, 1985*a*,*b*, 1986). Since a carbohydrate structure of AAT is similar to that of AFP (Mega *et al.*, 1980), LCA-reactive species of AAT is supposed to be a fucosylated fraction.

A characteristic feature of AAT is the multiple banding that is shown by isoelectric focusing. This banding is the result of microheterogeneity due to variations in its carbohydrate structure and a single amino acid substitution (Carell *et al.*, 1982). Therefore in order to investigate whether AAT phenotypes might change before and after the subsequent development of HCC, polyacrylamide gel slab isoelectric focusing was performed, using 15 pairs of serum samples. The result showed that all of the phenotypes were PiMM and that no change in phenotype was observed. In reference to the subtypes, PiM2M3, thought to be a predisposing factor to developing to chronic liver disease, was detected in 2 cases (13%) and this frequency was consistent with that of an earlier report (Miyake *et al.*, 1980).

The authors thank Associate professor Kazuhiko Miyake, The First Division, Department of Internal Medicine, Teikyo University School of Medicine, for his valuable experimental assistance.

### References

AOYAGI, Y., SUZUKI, Y., ISEMURA, M. & 6 others (1984). Differential reactivity of alpha-fetoprotein with lectins and evaluation of its usefulness in the diagnosis of hepatocellular carcinoma. *Gann*, **75**, 809.

AOYAGI, Y., SUZUKI, Y., ISEMURA, M. & 3 others (1985*a*). Fucosylated alpha-fetoprotein as marker of early hepatocellular carcinoma. *Lancet*, **ii**, 1353.

AOYAGI, Y., ISEMURA, M., SUZUKI, Y. & 3 others (1985*b*). Fucosylation of serum alpha-fetoprotein in patients with primary hepatocellular carcinoma. *Biochim. Biophys. Acta.*, **830**, 217.

AOYAGI, Y., ISEMURA, M., SUZUKI, Y. & 4 others (1986). Changes in fucosylation of alpha-fetoprotein on malignant transformation of liver cells. *Lancet*, **i**, 210.

ARNAUD, P., WILSON, G.B., KOISTINEN, J. & FUDENBERG, H.H. (1977). Immunofixation after electrofocusing; Improved method for specific detection of serum proteins with determination of isoelectric points I. immunofixation print technique for detection of alpha-1-protease inhibitor. *J. Immunol. Methods.*, **16**, 221.

BERG, N.O. & ERIKSSON, S. (1972). Liver disease in adults with alpha-1-antitrypsin deficiency. *N. Engl. J. Med.*, **287**, 1264.

BØG-HANSEN, T.C. (1973). Crossed immuno-affinity electrophoresis, an analytical method to predict the results of affinity chromatography. *Anal. Biochem.*, **56**, 480.

CARREL, R.W., JEPPSSON, J.O., LAURELL, C.B. & 4 others (1982). Structure and variation of human alpha-1-antitrypsin. *Nature*, **298**, 329.

ERIKSSON, S. & HAGERSTRAND, I. (1974). Cirrhosis and malignant hepatoma in alpha-1-antitrypsin deficiency. *Acta. Med. Scand.*, **195**, 451.

LAURELL, C.B. & ERIKSSON, S. (1963). The electrophoretic alpha-1-globulin pattern of serum in alpha-1-antitrypsin deficiency. *Scan. J. Clin. Lab. Invest.*, **15**, 132.

LIVER CANCER STUDY GROUP OF JAPAN (1983). *The General Rules for the Clinical and Pathological Study of Primary Liver Cancer*. p. 12. Kanehara Press Co. Ltd.: Tokyo.

MEGA, T., LUGAN, E. & YOSHIDA, A. (1980). Studies on the oligosaccharide chains of human alpha-1-protease inhibitor. *J. Biol. Chem.*, **255**, 4057.

MIYAKE, K., SUZUKI, H., OKA, H. & ODA, T. (1980). Phenotypes of alpha-1-antitrypsin in liver diseases. *Acta. Hepatol. Jpn.*, **21**, 1599.

ORDÓÑEZ, N.G. & MANNING, J.T. (1984). Comparison of alpha-1-antitrypsin and alpha-1-antichymotrypsin in hepatocellular carcinoma: An immunoperoxidase study. *Am. J. Gastroenterol.*, **79**, 959.

PALMER, P.E. & WOLFE, H.J. (1976). Alpha-1-antitrypsin deposition in primary hepatic carcinomas. *Arch. Pathol.*, **100**, 232.

PALMER, P.E., UCCL, A.A. & WOLFE, H.J. (1980). Expression of protein markers in malignant hepatoma. *Cancer*, **45**, 1424.

SHARP, H.L., BRIDGES, R.A., KRIVIT, W. & FREIR, E.F. (1969). Cirrhosis associated with alpha-1-antitrypsin deficiency; a previously unrecognized inherited disorder. *J. Lab. Clin. Med.*, **73**, 934.